# [1,1′-Bis(diphenyl­phosphino)cobalto­cenium-κ^2^ *P*,*P*′]dichloridoplatinum(II) hexa­fluorido­phosphate

**DOI:** 10.1107/S1600536809027925

**Published:** 2009-07-22

**Authors:** Jin-Tao Guan, Kai-Cheng Zhang, Zhi-Qun Dai

**Affiliations:** a College of Chemical and Environmental Engineering, Wuhan Polytechnic University, Wuhan 430023, People’s Republic of China

## Abstract

The title bimetallic compound, [PtCl_2_{Co(C_17_H_14_P)_2_}]PF_6_, was obtained by reaction of 1,1′-bis­(diphenyl­phosphino)cobalto­cenium hexa­fluorido­phosphate with bis­(acetonitrile)di­chloridoplatinum. The Pt^II^ ion is four-coordinated in a slightly distorted square-planar environment by two P atoms of the 1,1′-bis­(diphenyl­phosphino)cobaltocenium moiety and two Cl atoms. In the crystal structure, mol­ecules are linked by weak C—H⋯F and C—H⋯Cl hydrogen bonds.

## Related literature

For background to cobaltocene derivatives applied as catal­ysts, see: Brasse *et al.* (2000 ▶); Yu *et al.* (2007 ▶). For the structure of dichloro [1,1′-bis­(diphenyl­phosphino)ferrocene], see: Corain *et al.* (1989 ▶). For 1,1′-bis­(diphenyl­phosphino)cobalto­cenium tetra­fluoridoborate, see: Hou *et al.* (2007 ▶).
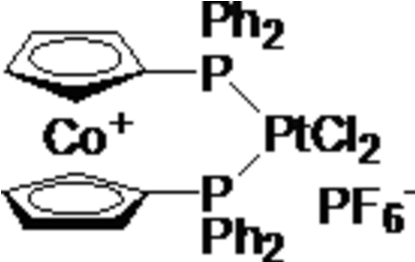


## Experimental

### 

#### Crystal data

[CoPt(C_17_H_14_P)_2_Cl_2_]PF_6_
*M* *_r_* = 968.39
Triclinic, 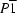

*a* = 8.9730 (1) Å
*b* = 10.8770 (1) Å
*c* = 19.7670 (1) Å
α = 94.9780 (1)°
β = 102.4230 (1)°
γ = 114.2320 (1)°
*V* = 1684.54 (3) Å^3^
*Z* = 2
Mo *K*α radiation
μ = 5.00 mm^−1^
*T* = 293 K
0.20 × 0.10 × 0.10 mm

#### Data collection

Bruker SMART CCD area-detector diffractometer
Absorption correction: multi-scan (*SADABS*; Sheldrick, 2004 ▶) *T*_min_ = 0.435, *T*_max_ = 0.635
17964 measured reflections
7506 independent reflections
6245 reflections with *I* > 2σ(*I*)
*R*_int_ = 0.080

#### Refinement

*R*[*F* ^2^ > 2σ(*F* ^2^)] = 0.040
*wR*(*F* ^2^) = 0.092
*S* = 1.00
7506 reflections
424 parameters
H-atom parameters constrained
Δρ_max_ = 1.98 e Å^−3^
Δρ_min_ = −1.19 e Å^−3^

### 

Data collection: *SMART* (Bruker, 2001 ▶); cell refinement: *SAINT* (Bruker, 2001 ▶); data reduction: *SAINT*; program(s) used to solve structure: *SHELXS97* (Sheldrick, 2008 ▶); program(s) used to refine structure: *SHELXL97* (Sheldrick, 2008 ▶); molecular graphics: *SHELXTL* (Sheldrick, 2008 ▶); software used to prepare material for publication: *SHELXTL*.

## Supplementary Material

Crystal structure: contains datablocks I, global. DOI: 10.1107/S1600536809027925/jh2085sup1.cif

Structure factors: contains datablocks I. DOI: 10.1107/S1600536809027925/jh2085Isup2.hkl

Additional supplementary materials:  crystallographic information; 3D view; checkCIF report

## Figures and Tables

**Table 1:** Hydrogen-bond geometry (Å, °)

*D*—H⋯*A*	*D*—H	H⋯*A*	*D*⋯*A*	*D*—H⋯*A*
C3—H3⋯F2^i^	0.98	2.32	3.190 (11)	148
C4—H4⋯Cl1^ii^	0.98	2.80	3.448 (7)	124
C7—H7⋯F5	0.98	2.55	3.513 (14)	168
C12—H12⋯F6^iii^	0.93	2.54	3.267 (11)	135
C14—H14⋯Cl2^iii^	0.93	2.79	3.497 (8)	134
C16—H16⋯Cl1	0.93	2.80	3.454 (7)	128
C22—H22⋯F5	0.93	2.51	3.311 (11)	145

Symmetry codes: (i) 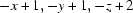; (ii) 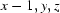; (iii) 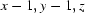.

**Fig. 1.**
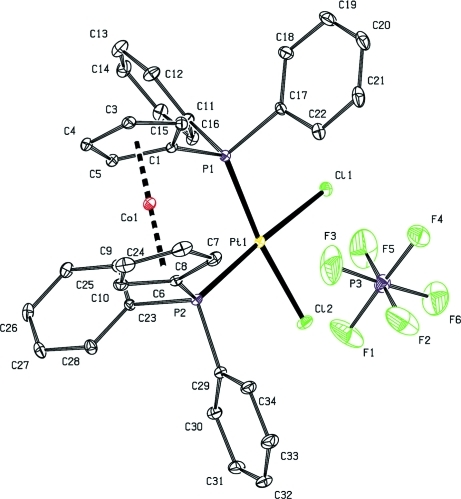
The molecular structure of (I), with atom labels and 50% probability displacement ellipsoids for non-H atoms. H atoms are omitted for clarity.

**Fig. 2.**
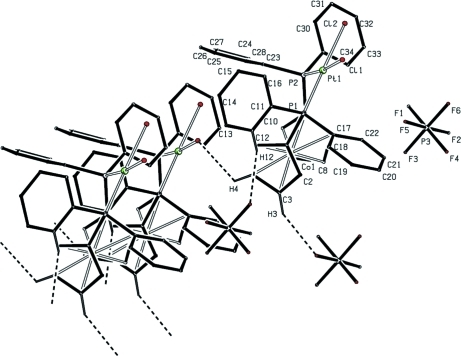
Hydrogen bonding interactions in (I).H atoms not involved in hydrogen bonding have been omitted.

## supplementary crystallographic information

### Comment

Cobaltocene derivatives have been applied as catalysts in cross-coupling reactions (Brasse *et al.*, 2000). As part of our investigations of new catalysts, we have focused our attention on cobaltocenium compounds. Some complexes, such as 1,1'-bis(diphenylphosphino)cobaltocenium tetrafluoridoborate, have been obtained and reported(Hou *et al.*, 2007). Herein, we report the structure of the title compound, (I) (Fig. 1), which is very similar to the complex dichloro [1,1'-bis(diphenylphosphino)ferrocene] (Corain *et al.*, 1989). The PtÎI^ metal ion is in an essentially square-planar environment defined by the two Cl atoms and the two P atoms. The angle between the plane P—Pt—P and the plane Cl—Pt—Cl is 4.0 (3)°. The bond angle of the two P atoms at the Pt atom is 100.64 (5)°, with the two Cl atoms it is 86.08 (5)°. The two substituted Cp rings are staggered and are essentially parallel with an interplanar angle of 3.62 (2)°. The crystal structure contains non-bonded C—H···F and C—H···Cl interactions (Fig.2).

### Experimental

A mixture of 1,1'-bis(dipehnylphosphio)cobaltocenium hexafluoridophosphate (70.2 mg, 0.1 mmol) and bis(acetonitrile)dichloroplatinum (34.8 mg, 0.1 mmol) in CH_2_Cl_2_(10 ml) was stirred for 4 h. The precipitate formed was separated by filtration and washed with Et_2_O and water, and dried under vacuum to give an yellow powder (85.2 mg). Crystals appropriate for data collection were obtained by slow evaporation from a CH_3_NO_2_ solution at 293 K.

### Refinement

All H atoms were placed in geometrically idealized positions and constrained to ride on their parent atoms with C—H = 0.93 Å; with *U*_iso_(H) = 1.2*U*_eq_(C).

### Crystal data

**Table:**

[CoPt(C_17_H_14_P)_2_Cl_2_]PF_6_	*Z* = 2
*M**_r_* = 968.39	*F*(000) = 940
Triclinic, *P*1	*D*_x_ = 1.909 Mg m^−^^3^
Hall symbol: -P 1	Mo *K*α radiation, λ = 0.71073 Å
*a* = 8.9730 (1) Å	Cell parameters from 7129 reflections
*b* = 10.8770 (1) Å	θ = 2.5–27.4°
*c* = 19.7670 (1) Å	µ = 5.00 mm^−^^1^
α = 94.9780 (1)°	*T* = 293 K
β = 102.4230 (1)°	Block, yellow
γ = 114.2320 (1)°	0.20 × 0.10 × 0.10 mm
*V* = 1684.54 (3) Å^3^	

### Data collection

**Table:**

Bruker SMART CCD area-detector diffractometer	7506 independent reflections
Radiation source: fine-focus sealed tube	6245 reflections with *I* > 2σ(*I*)
graphite	*R*_int_ = 0.080
φ and ω scans	θ_max_ = 27.5°, θ_min_ = 1.1°
Absorption correction: multi-scan (*SADABS*; Sheldrick, 2004)	*h* = −11→11
*T*_min_ = 0.435, *T*_max_ = 0.635	*k* = −13→13
17964 measured reflections	*l* = −25→25

### Refinement

**Table:**

Refinement on *F*^2^	Primary atom site location: structure-invariant direct methods
Least-squares matrix: full	Secondary atom site location: difference Fourier map
*R*[*F*^2^ > 2σ(*F*^2^)] = 0.040	Hydrogen site location: inferred from neighbouring sites
*wR*(*F*^2^) = 0.092	H-atom parameters constrained
*S* = 1.00	*w* = 1/[σ^2^(*F*_o_^2^) + (0.0412*P*)^2^ + 0.1224*P*] where *P* = (*F*_o_^2^ + 2*F*_c_^2^)/3
7506 reflections	(Δ/σ)_max_ = 0.027
424 parameters	Δρ_max_ = 1.98 e Å^−^^3^
0 restraints	Δρ_min_ = −1.19 e Å^−^^3^

### Special details

**Table:**

Geometry. All e.s.d.'s (except the e.s.d. in the dihedral angle between two l.s. planes) are estimated using the full covariance matrix. The cell e.s.d.'s are taken into account individually in the estimation of e.s.d.'s in distances, angles and torsion angles; correlations between e.s.d.'s in cell parameters are only used when they are defined by crystal symmetry. An approximate (isotropic) treatment of cell e.s.d.'s is used for estimating e.s.d.'s involving l.s. planes.
Refinement. Refinement of *F*^2^ against ALL reflections. The weighted *R*-factor *wR* and goodness of fit *S* are based on *F*^2^, conventional *R*-factors *R* are based on *F*, with *F* set to zero for negative *F*^2^. The threshold expression of *F*^2^ > σ(*F*^2^) is used only for calculating *R*-factors(gt) *etc*. and is not relevant to the choice of reflections for refinement. *R*-factors based on *F*^2^ are statistically about twice as large as those based on *F*, and *R*- factors based on ALL data will be even larger.

### Fractional atomic coordinates and isotropic or equivalent isotropic displacement parameters (Å^2^)

**Table:**

	*x*	*y*	*z*	*U*_iso_*/*U*_eq_	
Pt1	0.39843 (2)	0.173677 (19)	0.691223 (10)	0.02720 (8)	
C1	0.1265 (6)	0.0476 (5)	0.8029 (3)	0.0310 (11)	
C2	0.1575 (7)	0.0917 (6)	0.8766 (3)	0.0393 (13)	
H2	0.2662	0.1217	0.9124	0.047*	
C3	0.0069 (7)	0.0887 (6)	0.8896 (3)	0.0402 (14)	
H3	−0.0069	0.1162	0.9355	0.048*	
C4	−0.1191 (7)	0.0395 (6)	0.8243 (3)	0.0385 (13)	
H4	−0.2358	0.0284	0.8171	0.046*	
C5	−0.0475 (6)	0.0153 (5)	0.7704 (3)	0.0335 (12)	
H5	−0.1066	−0.0182	0.7199	0.040*	
C6	0.1783 (7)	0.3358 (5)	0.7545 (3)	0.0313 (11)	
C7	0.2929 (8)	0.3902 (6)	0.8247 (3)	0.0453 (15)	
H7	0.4113	0.4042	0.8385	0.054*	
C8	0.2032 (11)	0.4171 (7)	0.8706 (4)	0.061 (2)	
H8	0.2486	0.4506	0.9218	0.074*	
C9	0.0375 (11)	0.3835 (7)	0.8310 (4)	0.060 (2)	
H9	−0.0523	0.3899	0.8496	0.072*	
C10	0.0197 (8)	0.3319 (6)	0.7586 (3)	0.0462 (15)	
H10	−0.0830	0.3002	0.7191	0.055*	
C11	0.1492 (7)	−0.1487 (5)	0.7122 (3)	0.0350 (12)	
C12	0.0321 (8)	−0.2418 (6)	0.7413 (3)	0.0507 (16)	
H12	0.0269	−0.2148	0.7864	0.061*	
C13	−0.0751 (8)	−0.3725 (7)	0.7043 (4)	0.0605 (19)	
H13	−0.1526	−0.4341	0.7241	0.073*	
C14	−0.0672 (8)	−0.4123 (6)	0.6369 (4)	0.0574 (18)	
H14	−0.1420	−0.5001	0.6109	0.069*	
C15	0.0499 (8)	−0.3231 (7)	0.6084 (4)	0.0519 (16)	
H15	0.0562	−0.3515	0.5638	0.062*	
C16	0.1584 (7)	−0.1917 (6)	0.6453 (3)	0.0409 (14)	
H16	0.2376	−0.1316	0.6257	0.049*	
C17	0.4359 (7)	0.0302 (6)	0.8356 (3)	0.0373 (13)	
C18	0.4344 (8)	−0.0865 (7)	0.8568 (3)	0.0513 (16)	
H18	0.3546	−0.1725	0.8305	0.062*	
C19	0.5543 (11)	−0.0755 (10)	0.9187 (4)	0.073 (2)	
H19	0.5538	−0.1545	0.9335	0.088*	
C20	0.6699 (10)	0.0491 (11)	0.9567 (4)	0.074 (3)	
H20	0.7482	0.0555	0.9978	0.089*	
C21	0.6736 (8)	0.1670 (10)	0.9353 (4)	0.068 (2)	
H21	0.7538	0.2524	0.9622	0.081*	
C22	0.5600 (7)	0.1594 (7)	0.8746 (3)	0.0495 (16)	
H22	0.5650	0.2392	0.8594	0.059*	
C23	0.0296 (6)	0.1815 (5)	0.6113 (3)	0.0295 (11)	
C24	−0.0224 (7)	0.0419 (6)	0.5888 (3)	0.0367 (13)	
H24	0.0462	0.0008	0.6067	0.044*	
C25	−0.1782 (7)	−0.0353 (6)	0.5390 (3)	0.0433 (14)	
H25	−0.2142	−0.1290	0.5245	0.052*	
C26	−0.2803 (7)	0.0236 (7)	0.5108 (3)	0.0494 (17)	
H26	−0.3833	−0.0292	0.4769	0.059*	
C27	−0.2286 (8)	0.1602 (7)	0.5332 (3)	0.0497 (16)	
H27	−0.2978	0.2006	0.5146	0.060*	
C28	−0.0737 (7)	0.2407 (6)	0.5835 (3)	0.0419 (14)	
H28	−0.0399	0.3340	0.5982	0.050*	
C29	0.3237 (7)	0.4478 (6)	0.6492 (3)	0.0360 (12)	
C30	0.3094 (8)	0.4536 (6)	0.5787 (3)	0.0458 (15)	
H30	0.2458	0.3736	0.5445	0.055*	
C31	0.3895 (9)	0.5784 (8)	0.5587 (4)	0.0591 (19)	
H31	0.3807	0.5818	0.5112	0.071*	
C32	0.4826 (9)	0.6979 (7)	0.6099 (4)	0.063 (2)	
H32	0.5356	0.7819	0.5967	0.075*	
C33	0.4963 (9)	0.6918 (7)	0.6794 (4)	0.0629 (19)	
H33	0.5592	0.7720	0.7136	0.075*	
C34	0.4184 (8)	0.5689 (6)	0.6996 (3)	0.0513 (16)	
H34	0.4289	0.5664	0.7472	0.062*	
Cl1	0.57300 (18)	0.06097 (16)	0.69406 (8)	0.0458 (4)	
Cl2	0.5467 (2)	0.30193 (16)	0.61893 (9)	0.0502 (4)	
Co1	0.08481 (9)	0.21409 (8)	0.82014 (4)	0.03369 (17)	
P1	0.27781 (16)	0.02746 (14)	0.75901 (7)	0.0295 (3)	
P2	0.23250 (16)	0.28380 (13)	0.67663 (7)	0.0285 (3)	
F1	0.7096 (13)	0.6711 (9)	0.8492 (4)	0.209 (4)	
F4	0.9060 (8)	0.6276 (8)	0.9842 (3)	0.129 (2)	
F3	0.6545 (11)	0.6101 (15)	0.9389 (5)	0.255 (6)	
F5	0.7334 (12)	0.4950 (8)	0.8797 (7)	0.265 (6)	
F2	0.8821 (13)	0.7923 (8)	0.9449 (5)	0.230 (5)	
F6	0.9614 (11)	0.6650 (12)	0.8885 (4)	0.211 (5)	
P3	0.8058 (3)	0.6409 (2)	0.91318 (10)	0.0653 (5)	

### Atomic displacement parameters (Å^2^)

**Table:**

	*U*^11^	*U*^22^	*U*^33^	*U*^12^	*U*^13^	*U*^23^
Pt1	0.02182 (10)	0.02686 (12)	0.03064 (12)	0.00777 (8)	0.00884 (8)	0.00572 (8)
C1	0.030 (3)	0.028 (3)	0.037 (3)	0.012 (2)	0.013 (2)	0.012 (2)
C2	0.036 (3)	0.054 (4)	0.029 (3)	0.019 (3)	0.010 (2)	0.012 (3)
C3	0.045 (3)	0.048 (4)	0.036 (3)	0.022 (3)	0.021 (3)	0.018 (3)
C4	0.032 (3)	0.041 (3)	0.046 (3)	0.014 (2)	0.017 (3)	0.014 (3)
C5	0.033 (3)	0.032 (3)	0.032 (3)	0.010 (2)	0.010 (2)	0.005 (2)
C6	0.037 (3)	0.024 (3)	0.035 (3)	0.012 (2)	0.014 (2)	0.008 (2)
C7	0.046 (3)	0.031 (3)	0.038 (3)	0.001 (3)	0.008 (3)	−0.002 (3)
C8	0.094 (6)	0.037 (4)	0.043 (4)	0.016 (4)	0.028 (4)	0.000 (3)
C9	0.089 (6)	0.057 (5)	0.067 (5)	0.047 (4)	0.049 (4)	0.020 (4)
C10	0.052 (4)	0.044 (4)	0.059 (4)	0.029 (3)	0.026 (3)	0.024 (3)
C11	0.035 (3)	0.030 (3)	0.043 (3)	0.016 (2)	0.015 (2)	0.007 (2)
C12	0.060 (4)	0.032 (3)	0.059 (4)	0.014 (3)	0.030 (3)	0.008 (3)
C13	0.056 (4)	0.042 (4)	0.081 (5)	0.012 (3)	0.036 (4)	0.010 (4)
C14	0.045 (4)	0.029 (3)	0.085 (5)	0.008 (3)	0.015 (4)	−0.003 (3)
C15	0.049 (4)	0.046 (4)	0.055 (4)	0.017 (3)	0.017 (3)	−0.003 (3)
C16	0.034 (3)	0.038 (3)	0.048 (3)	0.012 (3)	0.014 (3)	0.006 (3)
C17	0.033 (3)	0.055 (4)	0.036 (3)	0.026 (3)	0.013 (2)	0.018 (3)
C18	0.055 (4)	0.060 (4)	0.057 (4)	0.036 (3)	0.023 (3)	0.027 (3)
C19	0.074 (5)	0.115 (8)	0.068 (5)	0.064 (5)	0.032 (4)	0.059 (5)
C20	0.052 (4)	0.142 (9)	0.049 (4)	0.057 (5)	0.018 (4)	0.042 (5)
C21	0.038 (4)	0.111 (7)	0.043 (4)	0.029 (4)	0.003 (3)	0.005 (4)
C22	0.041 (3)	0.061 (4)	0.047 (4)	0.026 (3)	0.009 (3)	0.005 (3)
C23	0.029 (3)	0.032 (3)	0.028 (3)	0.014 (2)	0.007 (2)	0.007 (2)
C24	0.034 (3)	0.038 (3)	0.039 (3)	0.016 (2)	0.011 (2)	0.008 (2)
C25	0.039 (3)	0.035 (3)	0.042 (3)	0.006 (3)	0.009 (3)	−0.006 (3)
C26	0.028 (3)	0.067 (5)	0.040 (3)	0.011 (3)	0.006 (3)	−0.003 (3)
C27	0.037 (3)	0.067 (5)	0.052 (4)	0.033 (3)	0.006 (3)	0.012 (3)
C28	0.038 (3)	0.040 (3)	0.049 (4)	0.023 (3)	0.007 (3)	0.002 (3)
C29	0.034 (3)	0.032 (3)	0.041 (3)	0.013 (2)	0.012 (2)	0.012 (2)
C30	0.051 (4)	0.042 (4)	0.048 (4)	0.020 (3)	0.019 (3)	0.015 (3)
C31	0.066 (4)	0.065 (5)	0.060 (4)	0.032 (4)	0.028 (4)	0.034 (4)
C32	0.058 (4)	0.041 (4)	0.092 (6)	0.017 (3)	0.029 (4)	0.036 (4)
C33	0.065 (5)	0.034 (4)	0.070 (5)	0.006 (3)	0.012 (4)	0.010 (3)
C34	0.051 (4)	0.037 (4)	0.050 (4)	0.007 (3)	0.008 (3)	0.013 (3)
Cl1	0.0384 (7)	0.0527 (9)	0.0572 (9)	0.0263 (7)	0.0193 (7)	0.0171 (7)
Cl2	0.0529 (9)	0.0433 (9)	0.0666 (10)	0.0201 (7)	0.0379 (8)	0.0221 (7)
Co1	0.0351 (4)	0.0350 (4)	0.0317 (4)	0.0140 (3)	0.0140 (3)	0.0054 (3)
P1	0.0275 (6)	0.0304 (7)	0.0321 (7)	0.0127 (6)	0.0101 (6)	0.0091 (6)
P2	0.0282 (6)	0.0246 (7)	0.0307 (7)	0.0088 (5)	0.0100 (6)	0.0055 (5)
F1	0.269 (10)	0.196 (9)	0.115 (5)	0.133 (8)	−0.088 (6)	−0.008 (5)
F4	0.119 (5)	0.172 (6)	0.091 (4)	0.062 (4)	0.013 (3)	0.053 (4)
F3	0.122 (6)	0.457 (19)	0.210 (9)	0.118 (9)	0.102 (7)	0.085 (10)
F5	0.172 (8)	0.088 (6)	0.445 (17)	0.039 (5)	−0.002 (10)	−0.082 (8)
F2	0.255 (11)	0.105 (6)	0.217 (9)	0.073 (6)	−0.119 (8)	−0.031 (6)
F6	0.165 (7)	0.350 (15)	0.138 (6)	0.101 (8)	0.091 (6)	0.074 (8)
P3	0.0571 (11)	0.0653 (14)	0.0542 (11)	0.0153 (10)	0.0056 (9)	0.0014 (10)

### Geometric parameters (Å, °)

**Table:**

Pt1—P2	2.2525 (14)	C15—H15	0.9300
Pt1—P1	2.2533 (13)	C16—H16	0.9300
Pt1—Cl2	2.3235 (13)	C17—C18	1.366 (8)
Pt1—Cl1	2.3473 (15)	C17—C22	1.403 (8)
C1—C2	1.424 (7)	C17—P1	1.828 (5)
C1—C5	1.432 (7)	C18—C19	1.403 (9)
C1—P1	1.833 (5)	C18—H18	0.9300
C1—Co1	2.010 (5)	C19—C20	1.346 (12)
C2—C3	1.416 (8)	C19—H19	0.9300
C2—Co1	2.025 (6)	C20—C21	1.374 (11)
C2—H2	0.9800	C20—H20	0.9300
C3—C4	1.409 (8)	C21—C22	1.367 (9)
C3—Co1	2.046 (5)	C21—H21	0.9300
C3—H3	0.9800	C22—H22	0.9300
C4—C5	1.415 (7)	C23—C28	1.381 (8)
C4—Co1	2.049 (5)	C23—C24	1.390 (8)
C4—H4	0.9800	C23—P2	1.816 (5)
C5—Co1	2.018 (5)	C24—C25	1.388 (8)
C5—H5	0.9800	C24—H24	0.9300
C6—C10	1.426 (8)	C25—C26	1.373 (9)
C6—C7	1.441 (8)	C25—H25	0.9300
C6—P2	1.823 (5)	C26—C27	1.361 (9)
C6—Co1	1.999 (5)	C26—H26	0.9300
C7—C8	1.420 (9)	C27—C28	1.394 (8)
C7—Co1	2.032 (5)	C27—H27	0.9300
C7—H7	0.9800	C28—H28	0.9300
C8—C9	1.400 (10)	C29—C30	1.381 (8)
C8—Co1	2.058 (6)	C29—C34	1.391 (8)
C8—H8	0.9800	C29—P2	1.818 (5)
C9—C10	1.441 (9)	C30—C31	1.390 (9)
C9—Co1	2.057 (7)	C30—H30	0.9300
C9—H9	0.9800	C31—C32	1.389 (10)
C10—Co1	2.011 (6)	C31—H31	0.9300
C10—H10	0.9800	C32—C33	1.364 (9)
C11—C16	1.394 (7)	C32—H32	0.9300
C11—C12	1.396 (7)	C33—C34	1.372 (8)
C11—P1	1.810 (6)	C33—H33	0.9300
C12—C13	1.369 (9)	C34—H34	0.9300
C12—H12	0.9300	F1—P3	1.512 (7)
C13—C14	1.392 (9)	F4—P3	1.550 (6)
C13—H13	0.9300	F3—P3	1.472 (7)
C14—C15	1.369 (9)	F5—P3	1.472 (8)
C14—H14	0.9300	F2—P3	1.511 (8)
C15—C16	1.377 (8)	F6—P3	1.507 (7)
			
P2—Pt1—P1	100.63 (5)	C25—C24—C23	119.1 (5)
P2—Pt1—Cl2	88.81 (5)	C25—C24—H24	120.5
P1—Pt1—Cl2	170.32 (5)	C23—C24—H24	120.5
P2—Pt1—Cl1	173.88 (5)	C26—C25—C24	121.5 (6)
P1—Pt1—Cl1	84.37 (5)	C26—C25—H25	119.3
Cl2—Pt1—Cl1	86.08 (5)	C24—C25—H25	119.3
C2—C1—C5	106.8 (4)	C27—C26—C25	119.1 (6)
C2—C1—P1	126.3 (4)	C27—C26—H26	120.5
C5—C1—P1	126.8 (4)	C25—C26—H26	120.5
C2—C1—Co1	69.9 (3)	C26—C27—C28	121.0 (6)
C5—C1—Co1	69.5 (3)	C26—C27—H27	119.5
P1—C1—Co1	128.0 (3)	C28—C27—H27	119.5
C3—C2—C1	108.9 (5)	C23—C28—C27	119.8 (6)
C3—C2—Co1	70.4 (3)	C23—C28—H28	120.1
C1—C2—Co1	68.8 (3)	C27—C28—H28	120.1
C3—C2—H2	125.5	C30—C29—C34	118.9 (5)
C1—C2—H2	125.5	C30—C29—P2	120.7 (4)
Co1—C2—H2	125.5	C34—C29—P2	120.2 (4)
C4—C3—C2	107.5 (5)	C29—C30—C31	120.3 (6)
C4—C3—Co1	70.0 (3)	C29—C30—H30	119.8
C2—C3—Co1	68.9 (3)	C31—C30—H30	119.8
C4—C3—H3	126.3	C32—C31—C30	119.7 (6)
C2—C3—H3	126.3	C32—C31—H31	120.2
Co1—C3—H3	126.3	C30—C31—H31	120.2
C3—C4—C5	109.0 (5)	C33—C32—C31	119.8 (6)
C3—C4—Co1	69.7 (3)	C33—C32—H32	120.1
C5—C4—Co1	68.5 (3)	C31—C32—H32	120.1
C3—C4—H4	125.5	C32—C33—C34	120.8 (7)
C5—C4—H4	125.5	C32—C33—H33	119.6
Co1—C4—H4	125.5	C34—C33—H33	119.6
C4—C5—C1	107.8 (5)	C33—C34—C29	120.4 (6)
C4—C5—Co1	70.8 (3)	C33—C34—H34	119.8
C1—C5—Co1	68.9 (3)	C29—C34—H34	119.8
C4—C5—H5	126.1	C6—Co1—C1	107.7 (2)
C1—C5—H5	126.1	C6—Co1—C10	41.7 (2)
Co1—C5—H5	126.1	C1—Co1—C10	134.9 (2)
C10—C6—C7	107.2 (5)	C6—Co1—C5	111.5 (2)
C10—C6—P2	127.8 (4)	C1—Co1—C5	41.7 (2)
C7—C6—P2	125.0 (4)	C10—Co1—C5	109.1 (2)
C10—C6—Co1	69.6 (3)	C6—Co1—C2	134.9 (2)
C7—C6—Co1	70.3 (3)	C1—Co1—C2	41.3 (2)
P2—C6—Co1	125.5 (3)	C10—Co1—C2	175.8 (2)
C8—C7—C6	108.0 (6)	C5—Co1—C2	69.1 (2)
C8—C7—Co1	70.7 (4)	C6—Co1—C7	41.9 (2)
C6—C7—Co1	67.8 (3)	C1—Co1—C7	111.7 (2)
C8—C7—H7	126.0	C10—Co1—C7	69.6 (3)
C6—C7—H7	126.0	C5—Co1—C7	142.6 (2)
Co1—C7—H7	126.0	C2—Co1—C7	109.3 (3)
C9—C8—C7	108.8 (6)	C6—Co1—C3	175.5 (2)
C9—C8—Co1	70.1 (4)	C1—Co1—C3	69.5 (2)
C7—C8—Co1	68.7 (3)	C10—Co1—C3	142.8 (2)
C9—C8—H8	125.6	C5—Co1—C3	68.9 (2)
C7—C8—H8	125.6	C2—Co1—C3	40.7 (2)
Co1—C8—H8	125.6	C7—Co1—C3	135.3 (2)
C8—C9—C10	108.2 (6)	C6—Co1—C4	142.7 (2)
C8—C9—Co1	70.1 (4)	C1—Co1—C4	69.0 (2)
C10—C9—Co1	67.5 (3)	C10—Co1—C4	113.4 (2)
C8—C9—H9	125.9	C5—Co1—C4	40.7 (2)
C10—C9—H9	125.9	C2—Co1—C4	68.0 (2)
Co1—C9—H9	125.9	C7—Co1—C4	175.4 (2)
C6—C10—C9	107.8 (6)	C3—Co1—C4	40.2 (2)
C6—C10—Co1	68.7 (3)	C6—Co1—C9	69.7 (2)
C9—C10—Co1	71.0 (4)	C1—Co1—C9	176.4 (2)
C6—C10—H10	126.1	C10—Co1—C9	41.5 (3)
C9—C10—H10	126.1	C5—Co1—C9	136.4 (3)
Co1—C10—H10	126.1	C2—Co1—C9	142.3 (3)
C16—C11—C12	119.0 (5)	C7—Co1—C9	68.2 (3)
C16—C11—P1	120.9 (4)	C3—Co1—C9	113.3 (2)
C12—C11—P1	120.0 (4)	C4—Co1—C9	111.4 (3)
C13—C12—C11	120.8 (6)	C6—Co1—C8	69.6 (2)
C13—C12—H12	119.6	C1—Co1—C8	142.3 (3)
C11—C12—H12	119.6	C10—Co1—C8	68.9 (3)
C12—C13—C14	119.4 (6)	C5—Co1—C8	175.9 (3)
C12—C13—H13	120.3	C2—Co1—C8	113.2 (3)
C14—C13—H13	120.3	C7—Co1—C8	40.6 (2)
C15—C14—C13	120.4 (6)	C3—Co1—C8	110.4 (2)
C15—C14—H14	119.8	C4—Co1—C8	136.2 (2)
C13—C14—H14	119.8	C9—Co1—C8	39.8 (3)
C14—C15—C16	120.5 (6)	C11—P1—C17	108.6 (3)
C14—C15—H15	119.8	C11—P1—C1	98.9 (2)
C16—C15—H15	119.8	C17—P1—C1	99.8 (2)
C15—C16—C11	119.9 (5)	C11—P1—Pt1	114.09 (18)
C15—C16—H16	120.0	C17—P1—Pt1	111.89 (17)
C11—C16—H16	120.0	C1—P1—Pt1	121.89 (17)
C18—C17—C22	119.8 (6)	C23—P2—C29	105.9 (2)
C18—C17—P1	123.0 (5)	C23—P2—C6	104.9 (2)
C22—C17—P1	117.2 (5)	C29—P2—C6	100.4 (2)
C17—C18—C19	119.5 (7)	C23—P2—Pt1	112.19 (18)
C17—C18—H18	120.2	C29—P2—Pt1	115.15 (18)
C19—C18—H18	120.2	C6—P2—Pt1	116.92 (18)
C20—C19—C18	120.1 (8)	F5—P3—F3	89.2 (7)
C20—C19—H19	119.9	F5—P3—F6	88.5 (6)
C18—C19—H19	119.9	F3—P3—F6	176.5 (7)
C19—C20—C21	120.8 (7)	F5—P3—F2	177.7 (7)
C19—C20—H20	119.6	F3—P3—F2	92.5 (7)
C21—C20—H20	119.6	F6—P3—F2	89.9 (6)
C22—C21—C20	120.4 (8)	F5—P3—F1	91.4 (6)
C22—C21—H21	119.8	F3—P3—F1	86.0 (6)
C20—C21—H21	119.8	F6—P3—F1	96.7 (6)
C21—C22—C17	119.4 (7)	F2—P3—F1	87.1 (5)
C21—C22—H22	120.3	F5—P3—F4	95.7 (6)
C17—C22—H22	120.3	F3—P3—F4	91.4 (5)
C28—C23—C24	119.5 (5)	F6—P3—F4	86.2 (4)
C28—C23—P2	120.8 (4)	F2—P3—F4	85.9 (4)
C24—C23—P2	119.7 (4)	F1—P3—F4	172.4 (5)
			
C5—C1—C2—C3	−0.8 (6)	C3—C2—Co1—C1	−120.5 (5)
P1—C1—C2—C3	−177.8 (4)	C3—C2—Co1—C10	−147 (3)
Co1—C1—C2—C3	59.1 (4)	C1—C2—Co1—C10	−27 (4)
C5—C1—C2—Co1	−59.9 (3)	C3—C2—Co1—C5	−81.5 (4)
P1—C1—C2—Co1	123.0 (4)	C1—C2—Co1—C5	39.0 (3)
C1—C2—C3—C4	1.5 (7)	C3—C2—Co1—C7	138.4 (4)
Co1—C2—C3—C4	59.6 (4)	C1—C2—Co1—C7	−101.2 (3)
C1—C2—C3—Co1	−58.1 (4)	C1—C2—Co1—C3	120.5 (5)
C2—C3—C4—C5	−1.6 (7)	C3—C2—Co1—C4	−37.6 (3)
Co1—C3—C4—C5	57.3 (4)	C1—C2—Co1—C4	82.9 (3)
C2—C3—C4—Co1	−58.9 (4)	C3—C2—Co1—C9	59.3 (6)
C3—C4—C5—C1	1.1 (6)	C1—C2—Co1—C9	179.7 (4)
Co1—C4—C5—C1	59.2 (3)	C3—C2—Co1—C8	94.8 (4)
C3—C4—C5—Co1	−58.1 (4)	C1—C2—Co1—C8	−144.7 (3)
C2—C1—C5—C4	−0.2 (6)	C8—C7—Co1—C6	119.8 (6)
P1—C1—C5—C4	176.8 (4)	C8—C7—Co1—C1	−147.6 (4)
Co1—C1—C5—C4	−60.4 (4)	C6—C7—Co1—C1	92.7 (4)
C2—C1—C5—Co1	60.2 (4)	C8—C7—Co1—C10	80.9 (5)
P1—C1—C5—Co1	−122.8 (4)	C6—C7—Co1—C10	−38.8 (3)
C10—C6—C7—C8	0.7 (6)	C8—C7—Co1—C5	176.0 (4)
P2—C6—C7—C8	−179.6 (4)	C6—C7—Co1—C5	56.2 (5)
Co1—C6—C7—C8	−59.5 (4)	C8—C7—Co1—C2	−103.4 (4)
C10—C6—C7—Co1	60.1 (4)	C6—C7—Co1—C2	136.8 (3)
P2—C6—C7—Co1	−120.1 (4)	C8—C7—Co1—C3	−65.3 (6)
C6—C7—C8—C9	−1.0 (7)	C6—C7—Co1—C3	174.9 (3)
Co1—C7—C8—C9	−58.7 (5)	C8—C7—Co1—C4	−50 (3)
C6—C7—C8—Co1	57.7 (4)	C6—C7—Co1—C4	−170 (3)
C7—C8—C9—C10	1.0 (8)	C8—C7—Co1—C9	36.4 (4)
Co1—C8—C9—C10	−56.9 (5)	C6—C7—Co1—C9	−83.4 (4)
C7—C8—C9—Co1	57.9 (5)	C6—C7—Co1—C8	−119.8 (6)
C7—C6—C10—C9	−0.1 (6)	C4—C3—Co1—C6	−132 (3)
P2—C6—C10—C9	−179.8 (4)	C2—C3—Co1—C6	−14 (3)
Co1—C6—C10—C9	60.5 (4)	C4—C3—Co1—C1	−81.5 (4)
C7—C6—C10—Co1	−60.6 (4)	C2—C3—Co1—C1	37.4 (3)
P2—C6—C10—Co1	119.7 (4)	C4—C3—Co1—C10	57.4 (6)
C8—C9—C10—C6	−0.6 (7)	C2—C3—Co1—C10	176.2 (4)
Co1—C9—C10—C6	−59.0 (4)	C4—C3—Co1—C5	−36.7 (3)
C8—C9—C10—Co1	58.5 (5)	C2—C3—Co1—C5	82.1 (4)
C16—C11—C12—C13	−1.5 (10)	C4—C3—Co1—C2	−118.9 (5)
P1—C11—C12—C13	175.0 (6)	C4—C3—Co1—C7	178.1 (4)
C11—C12—C13—C14	−0.2 (11)	C2—C3—Co1—C7	−63.0 (5)
C12—C13—C14—C15	1.8 (11)	C2—C3—Co1—C4	118.9 (5)
C13—C14—C15—C16	−1.7 (11)	C4—C3—Co1—C9	96.0 (4)
C14—C15—C16—C11	0.0 (10)	C2—C3—Co1—C9	−145.1 (4)
C12—C11—C16—C15	1.6 (9)	C4—C3—Co1—C8	138.9 (4)
P1—C11—C16—C15	−174.9 (5)	C2—C3—Co1—C8	−102.2 (4)
C22—C17—C18—C19	1.9 (9)	C3—C4—Co1—C6	174.5 (4)
P1—C17—C18—C19	−176.2 (5)	C5—C4—Co1—C6	53.3 (5)
C17—C18—C19—C20	−0.1 (10)	C3—C4—Co1—C1	82.6 (4)
C18—C19—C20—C21	−0.7 (11)	C5—C4—Co1—C1	−38.6 (3)
C19—C20—C21—C22	−0.4 (11)	C3—C4—Co1—C10	−146.3 (4)
C20—C21—C22—C17	2.1 (10)	C5—C4—Co1—C10	92.5 (4)
C18—C17—C22—C21	−2.9 (9)	C3—C4—Co1—C5	121.2 (5)
P1—C17—C22—C21	175.3 (5)	C3—C4—Co1—C2	38.0 (3)
C28—C23—C24—C25	−0.5 (8)	C5—C4—Co1—C2	−83.2 (4)
P2—C23—C24—C25	179.7 (4)	C3—C4—Co1—C7	−17 (3)
C23—C24—C25—C26	1.2 (8)	C5—C4—Co1—C7	−138 (3)
C24—C25—C26—C27	−1.3 (9)	C5—C4—Co1—C3	−121.2 (5)
C25—C26—C27—C28	0.7 (9)	C3—C4—Co1—C9	−101.3 (4)
C24—C23—C28—C27	0.0 (8)	C5—C4—Co1—C9	137.5 (4)
P2—C23—C28—C27	179.8 (4)	C3—C4—Co1—C8	−63.0 (5)
C26—C27—C28—C23	−0.1 (9)	C5—C4—Co1—C8	175.9 (4)
C34—C29—C30—C31	0.5 (9)	C8—C9—Co1—C6	−82.1 (4)
P2—C29—C30—C31	−175.5 (5)	C10—C9—Co1—C6	38.4 (4)
C29—C30—C31—C32	−0.8 (10)	C8—C9—Co1—C1	−126 (4)
C30—C31—C32—C33	0.7 (11)	C10—C9—Co1—C1	−5(4)
C31—C32—C33—C34	−0.3 (12)	C8—C9—Co1—C10	−120.5 (6)
C32—C33—C34—C29	0.0 (11)	C8—C9—Co1—C5	177.6 (3)
C30—C29—C34—C33	−0.1 (10)	C10—C9—Co1—C5	−61.9 (5)
P2—C29—C34—C33	175.9 (5)	C8—C9—Co1—C2	56.6 (6)
C10—C6—Co1—C1	139.1 (4)	C10—C9—Co1—C2	177.2 (4)
C7—C6—Co1—C1	−103.0 (4)	C8—C9—Co1—C7	−37.1 (4)
P2—C6—Co1—C1	16.5 (4)	C10—C9—Co1—C7	83.4 (4)
C7—C6—Co1—C10	117.9 (5)	C8—C9—Co1—C3	94.3 (4)
P2—C6—Co1—C10	−122.6 (5)	C10—C9—Co1—C3	−145.2 (4)
C10—C6—Co1—C5	95.0 (4)	C8—C9—Co1—C4	137.9 (4)
C7—C6—Co1—C5	−147.2 (4)	C10—C9—Co1—C4	−101.6 (4)
P2—C6—Co1—C5	−27.6 (4)	C10—C9—Co1—C8	120.5 (6)
C10—C6—Co1—C2	176.4 (4)	C9—C8—Co1—C6	82.4 (4)
C7—C6—Co1—C2	−65.7 (5)	C7—C8—Co1—C6	−38.2 (4)
P2—C6—Co1—C2	53.8 (5)	C9—C8—Co1—C1	175.2 (3)
C10—C6—Co1—C7	−117.9 (5)	C7—C8—Co1—C1	54.6 (6)
P2—C6—Co1—C7	119.5 (5)	C9—C8—Co1—C10	37.7 (4)
C10—C6—Co1—C3	−171 (3)	C7—C8—Co1—C10	−82.9 (4)
C7—C6—Co1—C3	−53 (3)	C9—C8—Co1—C5	−24 (3)
P2—C6—Co1—C3	66 (3)	C7—C8—Co1—C5	−144 (3)
C10—C6—Co1—C4	60.7 (5)	C9—C8—Co1—C2	−146.3 (4)
C7—C6—Co1—C4	178.6 (4)	C7—C8—Co1—C2	93.1 (4)
P2—C6—Co1—C4	−61.8 (5)	C9—C8—Co1—C7	120.6 (6)
C10—C6—Co1—C9	−38.2 (4)	C9—C8—Co1—C3	−102.4 (4)
C7—C6—Co1—C9	79.6 (4)	C7—C8—Co1—C3	137.0 (4)
P2—C6—Co1—C9	−160.8 (5)	C9—C8—Co1—C4	−64.5 (5)
C10—C6—Co1—C8	−80.8 (4)	C7—C8—Co1—C4	174.9 (4)
C7—C6—Co1—C8	37.1 (4)	C7—C8—Co1—C9	−120.6 (6)
P2—C6—Co1—C8	156.6 (5)	C16—C11—P1—C17	−111.8 (5)
C2—C1—Co1—C6	139.5 (3)	C12—C11—P1—C17	71.7 (5)
C5—C1—Co1—C6	−102.8 (3)	C16—C11—P1—C1	144.6 (5)
P1—C1—Co1—C6	18.6 (4)	C12—C11—P1—C1	−31.8 (6)
C2—C1—Co1—C10	177.4 (3)	C16—C11—P1—Pt1	13.7 (5)
C5—C1—Co1—C10	−64.9 (4)	C12—C11—P1—Pt1	−162.8 (5)
P1—C1—Co1—C10	56.4 (5)	C18—C17—P1—C11	−8.5 (5)
C2—C1—Co1—C5	−117.8 (4)	C22—C17—P1—C11	173.3 (4)
P1—C1—Co1—C5	121.3 (5)	C18—C17—P1—C1	94.4 (5)
C5—C1—Co1—C2	117.8 (4)	C22—C17—P1—C1	−83.8 (4)
P1—C1—Co1—C2	−120.9 (5)	C18—C17—P1—Pt1	−135.3 (4)
C2—C1—Co1—C7	95.0 (3)	C22—C17—P1—Pt1	46.5 (5)
C5—C1—Co1—C7	−147.2 (3)	C2—C1—P1—C11	126.1 (5)
P1—C1—Co1—C7	−25.9 (4)	C5—C1—P1—C11	−50.4 (5)
C2—C1—Co1—C3	−36.9 (3)	Co1—C1—P1—C11	−142.0 (4)
C5—C1—Co1—C3	80.9 (3)	C2—C1—P1—C17	15.4 (5)
P1—C1—Co1—C3	−157.8 (4)	C5—C1—P1—C17	−161.1 (5)
C2—C1—Co1—C4	−80.0 (3)	Co1—C1—P1—C17	107.2 (4)
C5—C1—Co1—C4	37.7 (3)	C2—C1—P1—Pt1	−108.2 (5)
P1—C1—Co1—C4	159.1 (4)	C5—C1—P1—Pt1	75.3 (5)
C2—C1—Co1—C9	−178 (4)	Co1—C1—P1—Pt1	−16.3 (4)
C5—C1—Co1—C9	−60 (4)	P2—Pt1—P1—C11	104.7 (2)
P1—C1—Co1—C9	62 (4)	Cl2—Pt1—P1—C11	−62.6 (4)
C2—C1—Co1—C8	60.2 (5)	Cl1—Pt1—P1—C11	−71.8 (2)
C5—C1—Co1—C8	178.0 (4)	P2—Pt1—P1—C17	−131.6 (2)
P1—C1—Co1—C8	−60.7 (5)	Cl2—Pt1—P1—C17	61.1 (4)
C9—C10—Co1—C6	−118.8 (6)	Cl1—Pt1—P1—C17	51.9 (2)
C6—C10—Co1—C1	−61.7 (5)	P2—Pt1—P1—C1	−13.8 (2)
C9—C10—Co1—C1	179.5 (4)	Cl2—Pt1—P1—C1	178.9 (3)
C6—C10—Co1—C5	−101.2 (3)	Cl1—Pt1—P1—C1	169.7 (2)
C9—C10—Co1—C5	140.0 (4)	C28—C23—P2—C29	−39.1 (5)
C6—C10—Co1—C2	−37 (4)	C24—C23—P2—C29	140.6 (4)
C9—C10—Co1—C2	−156 (3)	C28—C23—P2—C6	66.5 (5)
C6—C10—Co1—C7	39.0 (3)	C24—C23—P2—C6	−113.7 (4)
C9—C10—Co1—C7	−79.8 (4)	C28—C23—P2—Pt1	−165.6 (4)
C6—C10—Co1—C3	178.9 (4)	C24—C23—P2—Pt1	14.2 (4)
C9—C10—Co1—C3	60.0 (6)	C30—C29—P2—C23	−39.7 (5)
C6—C10—Co1—C4	−144.8 (3)	C34—C29—P2—C23	144.4 (5)
C9—C10—Co1—C4	96.4 (4)	C30—C29—P2—C6	−148.6 (5)
C6—C10—Co1—C9	118.8 (6)	C34—C29—P2—C6	35.5 (5)
C6—C10—Co1—C8	82.6 (4)	C30—C29—P2—Pt1	84.9 (5)
C9—C10—Co1—C8	−36.2 (4)	C34—C29—P2—Pt1	−91.0 (5)
C4—C5—Co1—C6	−148.5 (3)	C10—C6—P2—C23	−15.8 (5)
C1—C5—Co1—C6	92.7 (3)	C7—C6—P2—C23	164.4 (4)
C4—C5—Co1—C1	118.8 (4)	Co1—C6—P2—C23	74.8 (4)
C4—C5—Co1—C10	−104.0 (4)	C10—C6—P2—C29	93.9 (5)
C1—C5—Co1—C10	137.3 (3)	C7—C6—P2—C29	−85.8 (5)
C4—C5—Co1—C2	80.1 (3)	Co1—C6—P2—C29	−175.4 (3)
C1—C5—Co1—C2	−38.7 (3)	C10—C6—P2—Pt1	−140.8 (4)
C4—C5—Co1—C7	174.9 (4)	C7—C6—P2—Pt1	39.4 (5)
C1—C5—Co1—C7	56.1 (5)	Co1—C6—P2—Pt1	−50.2 (4)
C4—C5—Co1—C3	36.3 (3)	P1—Pt1—P2—C23	−77.79 (17)
C1—C5—Co1—C3	−82.5 (3)	Cl2—Pt1—P2—C23	100.09 (17)
C1—C5—Co1—C4	−118.8 (4)	Cl1—Pt1—P2—C23	66.8 (5)
C4—C5—Co1—C9	−65.8 (4)	P1—Pt1—P2—C29	160.9 (2)
C1—C5—Co1—C9	175.4 (3)	Cl2—Pt1—P2—C29	−21.2 (2)
C4—C5—Co1—C8	−44 (3)	Cl1—Pt1—P2—C29	−54.4 (5)
C1—C5—Co1—C8	−163 (3)	P1—Pt1—P2—C6	43.48 (19)
C3—C2—Co1—C6	178.5 (3)	Cl2—Pt1—P2—C6	−138.64 (19)
C1—C2—Co1—C6	−61.0 (4)	Cl1—Pt1—P2—C6	−171.9 (5)

### Hydrogen-bond geometry (Å, °)

**Table:**

*D*—H···*A*	*D*—H	H···*A*	*D*···*A*	*D*—H···*A*
C3—H3···F2^i^	0.98	2.32	3.190 (11)	148
C4—H4···Cl1^ii^	0.98	2.80	3.448 (7)	124
C7—H7···F5	0.98	2.55	3.513 (14)	168
C12—H12···F6^iii^	0.93	2.54	3.267 (11)	135
C14—H14···Cl2^iii^	0.93	2.79	3.497 (8)	134
C16—H16···Cl1	0.93	2.80	3.454 (7)	128
C22—H22···F5	0.93	2.51	3.311 (11)	145

Symmetry codes: (i) −*x*+1, −*y*+1, −*z*+2; (ii) *x*−1, *y*, *z*; (iii) *x*−1, *y*−1, *z*.
